# Grip Strength: A Useful Marker for Composite Hepatic Events in Patients with Chronic Liver Diseases

**DOI:** 10.3390/diagnostics10040238

**Published:** 2020-04-20

**Authors:** Kazunori Yoh, Hiroki Nishikawa, Hirayuki Enomoto, Yoshinori Iwata, Naoto Ikeda, Nobuhiro Aizawa, Takashi Nishimura, Hiroko Iijima, Shuhei Nishiguchi

**Affiliations:** 1 Division of Hepatobiliary and Pancreatic disease, Department of Internal Medicine, Hyogo College of Medicine, Nishinomiya, Hyogo 663-8501, Japan; mm2wintwin@ybb.ne.jp (K.Y.); enomoto@hyo-med.ac.jp (H.E.); yo-iwata@hyo-med.ac.jp (Y.I.); nikeneko@hyo-med.ac.jp (N.I.); nobu23hiro@yahoo.co.jp (N.A.); tk-nishimura@hyo-med.ac.jp (T.N.); hiroko-i@hyo-med.ac.jp (H.I.); nishiguc@hyo-med.ac.jp (S.N.); 2 Center for Clinical Research and Education, Hyogo College of Medicine, Nishinomiya, Hyogo 663-8501, Japan

**Keywords:** chronic liver disease, composite hepatic events, grip strength, muscle mass, quality of life, predictor

## Abstract

Here we sought to clarify the prognostic impact of sarcopenia-related markers (grip strength (GS), muscle mass using bioimpedance analysis and patient quality of life as assessed by the 36-Item Short-Form Health Survey (SF36)) in patients with chronic liver diseases (CLDs, *n* = 411; 160 liver cirrhosis patients; median age, 64 years) on the incidence of composite hepatic events (CHEs). A GS decrease was defined as <26 kg in men and <18 kg in women, while a skeletal muscle mass index (SMI) decrease was defined as <7.0 kg/m^2^ in men and <5.7 kg/m^2^ in women based on the current guidelines. The physical and metal component summary scores on the SF36 were also included into the analysis. Sixty-two patients (15.1%) had the first incidence of CHEs. The three-year cumulative incidence rates of CHEs in patients with GS decrease or non-decrease were 24.51% and 12.44% (*p* = 0.0057). The three-year cumulative incidence rates of CHEs in patients with an SMI decrease or non-decrease were 19.65% and 12.99% (*p* = 0.0982). Multivariate analysis revealed that GS decrease (*p* = 0.0350) and prothrombin time (*p* = 0.0293) were significantly associated with the incidence of CHEs. In conclusion, GS can be an independent predictor for CHE development in patients with CLDs.

## 1. Introduction

Skeletal muscle is crucial for mobility, regulating the metabolism of glucose and fats, cardiopulmonary function, immune function, cytokine balance, and activities of daily life [1,2]. Sarcopenia, as defined by decreased muscle strength and a muscle mass decline, is a common syndrome in patients with chronic liver diseases (CLDs) due to the disease burden itself (i.e., secondary sarcopenia) [3,4,5,6]. Physical activity and quality of life (QOL) are also likely to be impaired in CLD patients with sarcopenia due to skeletal muscle mass loss and muscle contractile dysfunction [7,8]. Lifestyle and physical health in early life can be long-term determinants of a muscle strength decline [9]. Several previous reports highlighted the importance of improving QOL in patients with CLDs [10,11,12,13,14]. We previously demonstrated that grip strength (GS) was closely linked to health-related QOL as assessed by the 36-Item Short-Form Health Survey (SF36) in patients with CLDs [15]. A recent meta-analysis reported that sarcopenia may be associated with higher mortality in CLD patients [3]. Combined Model for End-Stage Liver Disease (MELD) and sarcopenia are associated with improved predictability for mortality in patients with liver cirrhosis (LC), mainly in those with low MELD scores [16]. Sarcopenia has been gaining much attention these days in numerous chronic diseases and malignancies as well as in elderly persons [4,5,17,18,19]. However, which of the sarcopenia-related factors (GS, muscle mass, or patient QOL) is most likely associated with clinical outcomes in CLD patients remains unsolved. Elucidating this point is of clinical importance for establishing the appropriate interventional strategies in CLD patients.

Considering clinical outcomes in patients with CLDs, composite hepatic events (CHEs), which involve hepatic decompensation, new-onset or deterioration of ascites, variceal bleeding, acute-on-chronic liver failure, hepatic encephalopathy (HE), and hepatocellular carcinoma (HCC), have been extensively used to assess the outcomes of patients with CLDs [20,21,22,23,24]. These events are well-known predictors for survival in CLDs [25,26,27,28,29,30]. Composite study endpoints are preferentially used, mainly in the field of cardiac diseases [31,32,33]. However, the prognostic impact of sarcopenia-related markers in patients with CLDs on CHEs remains unclear. In this study, we sought to clarify those essential research issues.

## 2. Patients and Methods

### 2.1. Patients

A total of 411 individuals with CLD who were admitted to Hyogo College of Medicine Hospital, Japan, between December 2013 and March 2019 were consecutively enrolled. Data for muscle strength as evaluated by GS, bioimpedance analysis (BIA; data for muscle mass), and SF36 scores were collected. There was no clear evidence of HCC in any patients on baseline radiology.

During the follow-up period, hematological and radiologic tests for identifying HCC incidence or liver-related complications were performed every 3–6 months. LC or non-LC was determined according to pathological data, imaging findings (presence of liver deformity, splenomegaly, or varices) and/or laboratory data [34,35,36]. In cases of a serum albumin level falling to less than 3.5 g/dL, nutritional interventions such as branched-chain amino acid therapies were considered [37,38]. In cases of hepatitis C virus (HCV) or hepatitis B virus (HBV), antiviral treatments were also considered [37]. Alcoholic liver disease patients were instructed to stop all alcohol intake. For patients with other liver disease etiologies, the most adequate intervention for each underlying liver disease was provided. The diagnosis of any CHE was determined based on the current guidelines; in cases of CHE incidence, an appropriate therapy was provided according to the current guidelines [27,29,30,39,40]. No patients underwent liver transplantation during the observation period.

The current study protocol conformed to the 1975 Helsinki Declaration, and ethical approval was obtained from the institutional review board in the Hyogo College of Medicine Hospital. All personal information was protected.

### 2.2. GS and Skeletal Muscle Mass Index

Baseline GS and skeletal muscle mass index (SMI) using BIA (calculated by appendicular muscle mass divided by height squared (kg/m^2^)) were tested based on the current guidelines [19]. A GS decrease was defined as <26 kg in men and <18 kg in women [19]. An SMI decrease was defined as <7.0 kg/m^2^ in men and <5.7 kg/m^2^ in women [19].

### 2.3. Primary Outcome Measure of This Study

The primary endpoint of this study was the first incidence of CHEs [20,21,22,23,24]. CHEs included the incidence of hepatic decompensation, new-onset or deterioration of ascites, variceal bleeding, acute-on-chronic liver failure, HE, or HCC. During the observation period, any CHE was registered. Patients were followed from the baseline measurement of sarcopenia-related markers (i.e., GS, SMI, and SF36) until the first incidence of CHEs, death, or until the end of follow-up.

### 2.4. Physical Component Summary Score and Mental Component Summary Score on the SF36

The study subjects were asked to complete the Japanese version of the SF36, a globally accepted assessment tool for patient QOL [41,42]. The SF36 consists of a total of 36 items classified into eight item scales: physical functioning (PF), role physical (RP), bodily pain (BP), general health perception (GH), vitality (VT), social functioning (SF), role emotion (RE), and mental health (MH) [43]. The physical component summary (PCS summary score for PF, RP, BP, and GH) and the mental component summary (MCS summary score for VT, SF, RE, and MH) were analyzed in this study [43]. A higher PCS and higher MCS indicate a favorable health status. Patients with overt encephalopathy were not included in this study.

### 2.5. Statistical Analyses

In the univariate analyses of factors related to the incidence of CHEs, the median value for each factor was chosen to divide the study cohort into the two categorical groups and was then treated as a nominal variable. Factors with values of *p* < 0.01 on univariate analysis were entered into the multivariate Cox hazard model considering the number of CHEs. Survival curves were made by the Kaplan–Meier method and compared by the log-rank test. Data were shown as median value with interquartile range (IQR). The level of statistical significance was set at *p* < 0.05 by the statistical analysis software (JMP 14 (SAS Institute Inc., Cary, NC)).

## 3. Results

### 3.1. Baseline Characteristics

The demographic and clinical characteristics of the analyzed subjects (*n* = 411) are presented in Table 1. The study cohort included 199 men and 212 women with the median age (IQR) of 64 (54, 71) years. The median follow-up duration was 3.55 years. Regarding liver disease etiologies, patients were predominantly HCV (258/411 (62.8%)). LC was seen in 160 patients (38.9%). Among the LC patients, 121 were Child–Pugh A, 33 were Child–Pugh B, and 6 were Child–Pugh C. The median (IQR) GS values were 34.3 kg (28.65, 41.2 kg) in men and 20.95 kg (17.5375, 23.875 kg) in women. Among the men, 29 patients (14.6%) had a GS decrease, while among the women, 60 patients (28.3%) had a GS decrease as defined by the current Japanese criteria [19]. The median (IQR) SMI values were 7.50 kg/m^2^ (6.97, 7.98 kg/m^2^) in men and 5.92 kg/m^2^ (5.46, 6.46 kg/m^2^) in women. Among the men, 52 patients (26.1%) had an SMI decrease, while among the men, 73 patients (34.4%) had an SMI decrease as defined by the current Japanese criteria [19]. The median (IQR) PCS and MCS were 51.1 (41.6, 54.3) and 52.3 (44.2, 58.7), respectively.

### 3.2. Cumulative Incidences of CHEs for All Cases

**Figure 1:**
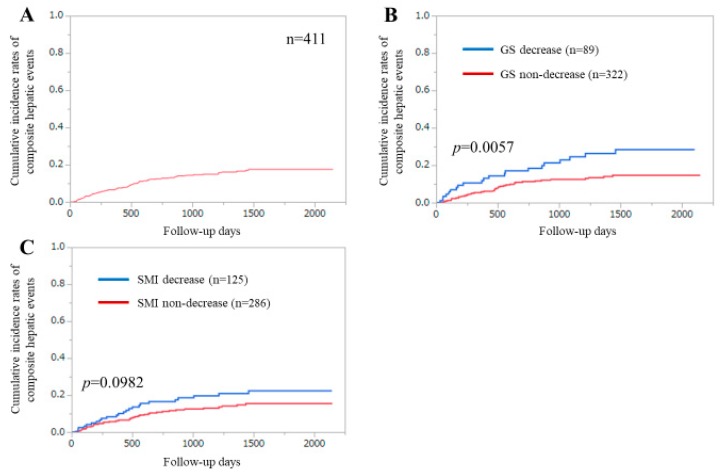
(**A**) Cumulative incidence of composite hepatic events (CHEs) for all cases (*n* = 411). (**B**) Cumulative incidence of CHEs stratified by grip strength (GS). (**C**) Cumulative incidence of CHEs stratified by skeletal muscle mass index (SMI).

The first incidence of CHEs was our primary outcome measure. During the follow-up period, 62 patients (15.1%) had the first incidence of CHEs. Of them, HCC development accounted for 54.8% (34 patients). None of the patients underwent liver transplantation. The 1-, 3-, and 5-year cumulative incidences of CHEs were 6.76%, 14.98%, and 17.55%, respectively (Figure 1A).

### 3.3. Cumulative Incidences of CHEs for All Cases Stratified by GS or SMI

The 1-, 3-, and 5-year cumulative incidences of CHEs were 10.57%, 24.51%, and 28.44% in the GS decrease group and 5.74%, 12.44%, and 14.62% in the GS non-decrease group (*p* = 0.0057) (Figure 1B).

The 1-, 3-, and 5-year cumulative incidences of CHEs were 8.33%, 19.65%, and 22.34% in the SMI decrease group and 6.09%, 12.99%, and 15.47% in the SMI non-decrease group (*p* = 0.0982) (Figure 1C).

**Figure 2:**
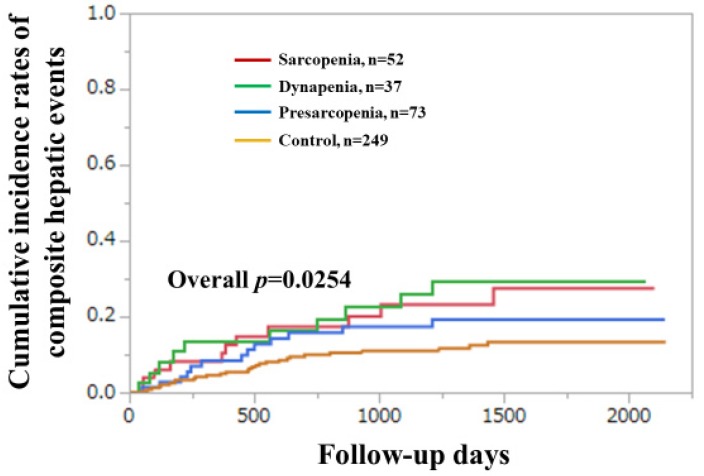
Cumulative incidence of composite hepatic events (CHEs) among the four groups. Sarcopenia indicates grip strength (GS) decrease and skeletal muscle mass index (SMI) decrease. Dynapenia indicates a GS decrease and SMI indicates a non-decrease. Presarcopenia indicates a GS non-decrease and an SMI decrease. Control indicates a GS non-decrease and SMI indicates a non-decrease.

We further divided the study subjects into four groups: (1) patients with GS decrease and SMI decrease (sarcopenia, *n* = 52); (2) patients with GS decrease and SMI non-decrease (dynapenia, *n* = 37); (3) patients with GS non-decrease and SMI decrease (presarcopenia, *n* = 73); and (4) patients with GS non-decrease and SMI non-decrease (control, *n* = 249). The cumulative incidences of CHEs among the four groups reached significance (*p* = 0.0254) (Figure 2).

### 3.4. Uni- and Multivariate Analyses of Factors Linked to the Incidence of CHEs

The univariate analysis of factors linked to the incidence of CHEs revealed that seven had values of *p* < 0.01: age ≥64 years (*p* < 0.0001), GS decrease (*p* = 0.0057), serum albumin ≥4.2 g/dL (*p* < 0.0001), prothrombin time (PT) ≥90.0% (*p* < 0.0001), platelet count ≥16.6 × 10^4^/mm^3^ (*p* < 0.0001), aspartate aminotransferase ≥27 IU/L (*p* = 0.0008), and branched-chain amino acid to tyrosine ratio ≥5.64 (*p* < 0.0001) (Table 2). Multivariate analysis of the seven factors showed that GS decrease (*p* = 0.0350) and PT ≥90.0% (*p* = 0.0293) were significant factors associated with the incidence of CHEs (Table 2). Hazard ratios (HRs) and 95% confidence intervals (CIs) for these items are shown in Table 2. The presence of LC was not included in the analysis because it can be a confounding factor for liver functional parameters such as total bilirubin, serum albumin, and PT.

### 3.5. Subgroup Analysis 1. Cumulative Incidences of CHEs for LC Cases and Non-LC Cases Stratified by GS and SMI

**Figure 3:**
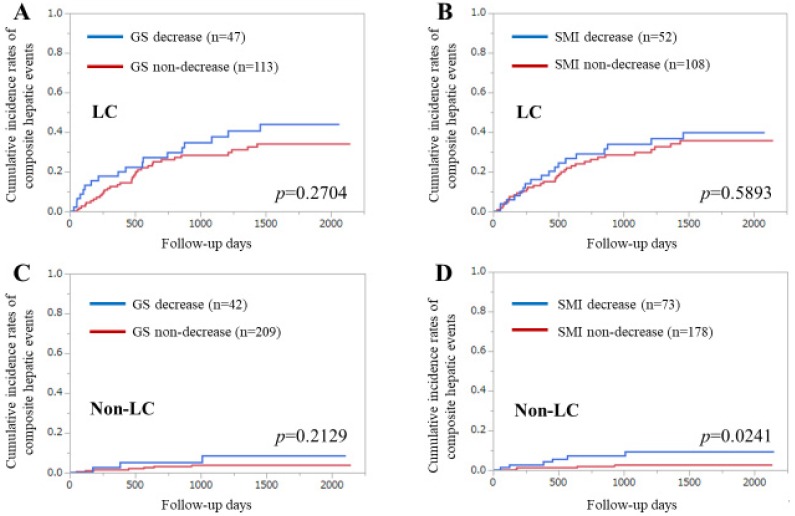
Subgroup analyses by liver cirrhosis (LC) status. (**A**) Cumulative incidence of composite hepatic events (CHEs) stratified by grip strength (GS) in LC patients. (**B**) Cumulative incidences of CHEs stratified by skeletal muscle mass index (SMI) in LC patients. (**C**) Cumulative incidences of CHEs stratified by GS in non-LC patients. (**D**) Cumulative incidences of CHEs stratified by SMI in non-LC patients.

Among the LC cases (*n* = 160), the difference in the cumulative incidences of CHEs between the GS decrease and non-decrease groups did not reach significance (*p* = 0.2704), nor did that between the SMI decrease and non-decrease groups (*p* = 0.5893) (Figure 3A,B).

Among the non-LC cases (*n* = 252), the difference in the cumulative incidences of CHEs between the GS decrease and GS non-decrease groups did not reach significance (*p* = 0.2129), while that between the SMI decrease and non-decrease groups reached significance (*p* = 0.0241) (Figure 3C,D).

### 3.6. Subgroup Analysis 2. Cumulative Incidence Rates of CHEs for Male and Female Cases Stratified by GS and SMI

**Figure 4:**
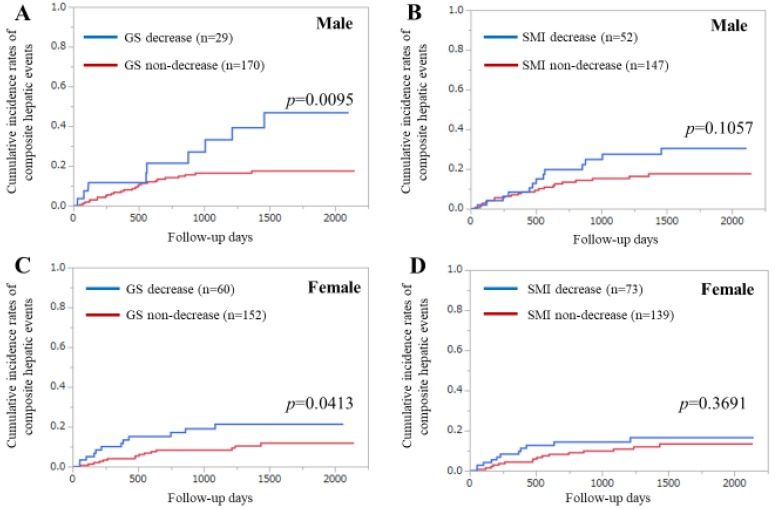
Subgroup analyses according to gender. (**A**) Cumulative incidence of CHEs stratified by GS in male. (**B**) Cumulative incidence of CHEs stratified by SMI in male. (**C**) Cumulative incidence of CHEs stratified by GS in female. (**D**) Cumulative incidence of CHEs stratified by SMI in female.

Among the men (*n* = 199), the difference in the cumulative incidences of CHEs between the GS decrease and non-decrease groups reached significance (*p* = 0.0095), while that between the SMI decrease and non-decrease groups was not significant (*p* = 0.1057) (Figure 4A,B).

Among the women (*n* = 212), the difference in the cumulative incidences of CHEs between the GS decrease and non-decrease groups reached significance (*p* = 0.0413), while that between the SMI decrease and non-decrease groups did not reach significance (*p* = 0.3691) (Figure 4C,D).

### 3.7. Subgroup Analysis 3. Cumulative Incidence Rates of CHEs for Elderly Cases and Younger Cases Stratified by GS and SMI

**Figure 5:**
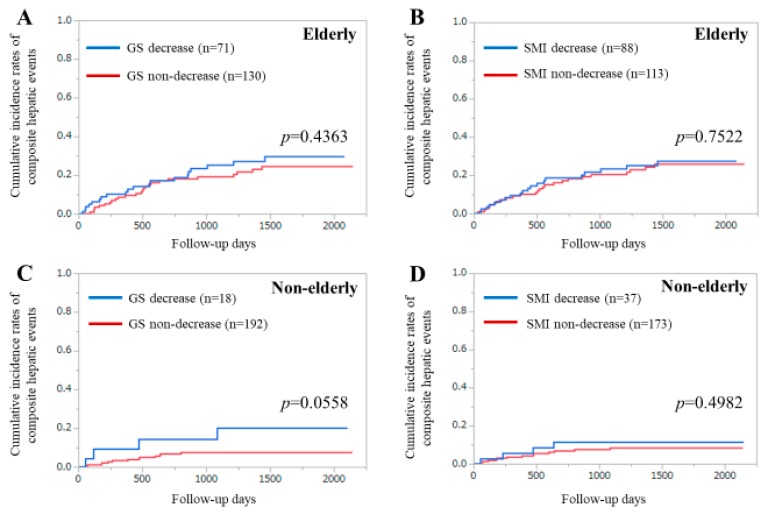
Subgroup analyses by age. (**A**) Cumulative incidence of composite hepatic events (CHEs) stratified by grip strength (GS) in patients aged 65 years or older. (**B**) Cumulative incidence of CHEs stratified by skeletal muscle mass index (SMI) in patients aged 65 years or older. (**C**) Cumulative incidence of CHEs stratified by GS in patients aged less than 65 years. (**D**) Cumulative incidence of CHEs stratified by SMI in patients aged less than 65 years.

Among the elderly cases (≥ 65 years, *n* = 201), the difference in the cumulative incidences of CHEs between the GS decrease and non-decrease groups was not significant (*p* = 0.4363), while that between the SMI decrease and non-decrease groups was also not significant (*p* = 0.7522) (Figure 5A,B).

In younger cases (< 65 years, *n* = 210), the difference in the cumulative incidences of CHEs between the GS decrease and non-decrease groups tended to be significant (*p* = 0.0558), while that between the SMI decrease and non-decrease groups did not reach significance (*p* = 0.4982) (Figure 5C,D).

### 3.8. Additional Analyses Using Different Cutoff Point of GS in Men (<28 kg)

**Figure 6:**
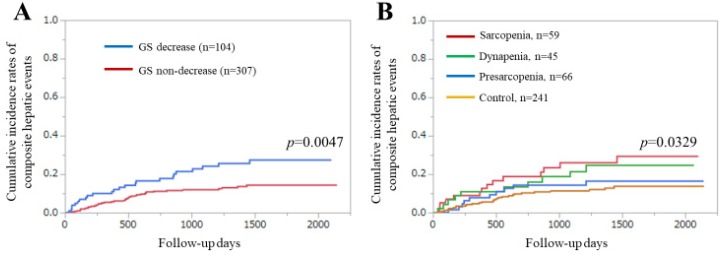
(**A**) Cumulative incidence of composite hepatic events (CHEs) stratified by grip strength (GS) for all cases (different cutoff point of GS in men, <28 kg). (**B**) Cumulative incidence of CHEs among the four groups. Sarcopenia indicates a GS decrease and skeletal muscle mass index (SMI) decrease. Dynapenia indicates a GS decrease and an SMI non-decrease. Presarcopenia indicates a GS non-decrease and an SMI decrease. Control indicates a GS non-decrease and an SMI non-decrease.

The Asian Working Group for Sarcopenia (AWGS) group recently revised the cutoff point of GS in men from <26 to <28 kg (that in women remained <18 kg) [44]. Thus, we further analyzed the cohort using this revised cutoff point. The GS decreased in men increased from 29 to 44. The difference in the cumulative incidences of CHEs between the GS decrease and non-decrease groups remained significant (*p* = 0.0047) (Figure 6A). The difference in the cumulative incidences of CHEs among the four groups (sarcopenia (*n* = 59), dynapenia (*n* = 45), presarcopenia (*n* = 66), and control (*n* = 241)) also remained significant (*p* = 0.0329) (Figure 6B). Multivariate analysis of the seven factors determined that a GS decrease (*p* = 0.0460) and PT (*p* = 0.0310) were significant factors associated with the incidence of CHEs (Table 3). HRs and 95% CIs for these items are shown in Table 3.

## 4. Discussion

To the best of our knowledge, this is the first study to elucidate the effect of sarcopenia-related factors on CHEs in patients with CLDs. Liver carcinogenesis, liver functional reserve-related factors, and portal hypertension-related factors appear to be well-established predictors for survival in CLDs [25,26,27,28,29,30]. Thus, we believe that the incidence of CHEs is an appropriate primary outcome measure. The decrease in patient QOL in CLDs tends to be frequently overlooked or unrecognized because patient QOL in CLDs can be compromised. In this study, SF36 data (PCS and MCS) were included in the analysis. One of major strengths in this study is its large sample size with GS, SMI, and SF36 data all available.

In our results, GS was an independent predictor for the incidence of CHEs in the multivariate analysis. When the GS cutoff in men was changed to <28 kg, similar results were obtained. In several subgroups, significant impacts of the GS decline on CHEs were found. Collectively, these results indicated that GS can be a useful marker for the incidence of CHEs in patients with CLDs. When we looked at the Kaplan–Meier curves shown in Figure 2 and Figure 6B, we noticed that curves of the sarcopenia or dynapenia group were almost constantly above those of the presarcopenia or control group, which supports the impact of GS on CHEs in our analysis. Because a reduced muscle mass generally precedes a reduced muscle strength, dynapenia may have unique characteristics that can involve serious negative consequences [45]. Appropriate interventional strategies for CLD patients with GS decline should be recommended. While SMI was not a significant predictor of the incidence of CHEs, muscle mass appears to play a relatively minor role on the incidence of CHEs compared with muscle function.

A previous large observational study (*n* = 139,691) demonstrated that GS measurement is a simple, inexpensive risk-stratifying method for all-cause mortality, cardiovascular-related death, and cardiovascular disease [46]. In addition, a greater GS was significantly linked to a reduced risk of cancer mortality [47]. A previous report demonstrated that GS should be considered a vital sign useful for screening older as well as middle-aged adults [48]. A previous meta-analysis reported that objective measures of physical function (i.e., GS, walking speed, chair rising, and standing balance times) were predictive factors for all-cause mortality in elderly community-dwelling populations [49]. As muscle function reacts early to nutrition deprivation, GS can be a reliable marker for nutrition status [50,51]. These observations may be linked to our current results. Contrary to our expectations, PCS in the SF36 was not an independent predictor in our analysis, although it was significant in the univariate analysis. SF36 is a subjective assessment method of patient QOL and may have affected our results [43]. However, we believe that our results do not deny the importance of maintaining patient QOL in the management of CLDs.

The cutoff point of GS in men was recently revised from <26 to <28 kg (that in women remains <18kg) in the revised AWGS criteria [44]. About three decades have passed since sarcopenia was proposed by Rosenberg in 1989 as a condition in which skeletal muscle mass decreases with age [52]. Muscle atrophy undoubtedly develops with age [45]. For the past several decades, sarcopenia research has largely focused on muscle size [19]. However, studies in this field have progressed and emphasis has been placed on the qualitative evaluation of muscle and physical function as well as muscle mass. The diagnosis of sarcopenia has been made with many diseases and has significantly attracted attention, and sarcopenia has been recognized as an independent disease entity. Significant impacts of GS on CHEs in cases with a cutoff of <26 kg in men and <28 kg in men in the multivariate analyses suggests the prognostic robustness of GS on the incidence of CHEs in CLD patients. While reference values of GS may be revised in the future, it is necessary to re-examine the clinical significance of sarcopenia-related factors accordingly.

Several limitations must be acknowledged to this study. First, this single-center observational retrospective study should be acknowledged. Second, patients with massive ascites at baseline who potentially have a higher incidence of liver disease–related events were excluded due to a lack of BIA results, creating bias. Third, our follow-up period (median 3.55 years) was relatively short for the analysis, especially in non-LC patients because the number of expected CHEs is low in non-LC patients. Fourth, our cohort had highly heterogeneous patient populations including various liver disease etiologies and various stages of liver function, and several subgroup analyses had small case number of cohort (e.g., non-elderly GS decrease (*n* = 18)). However, we believe the multivariate analysis reduced these biases. Finally, the impact of sarcopenia-related factors on overall survival (OS) was not analyzed in this study (beyond the scope of the current analysis). The impact of GS or other sarcopenia-related factors on OS in patients with CLDs should be fully examined in future studies. Consequently, caution must be exercised in the interpretation of our study data. Despite these limitations, our study results suggest that a GS decrease in CLDs was closely associated with the incidence of CHEs. In conclusion, GS can be an independent predictor for CHE development in patients with CLDs. Clinicians should be aware of the possibility of CHEs in CLD patients with a decreased GS.

## Figures and Tables

**Table 1:** Baseline characteristics (*n* = 411).

Variable	Number or
	Median Value (IQR)
Age (years)	64 (54, 71)
Sex, male/female	199/212
Body mass index (kg/m^2^)	22.6 (20.5, 25.5)
Liver disease etiology	258/59/8/86
(HCV/HBV/HCV and HBV/NBNC)	
Grip strength (kg, male)	34.3 (28.65, 41.2)
Grip strength (kg, female)	20.95 (17.5375, 23.875)
Presence of LC, yes/no	160/251
Total bilirubin (mg/dL)	0.8 (0.6, 1.0)
Serum albumin (g/dL)	4.2 (3.9, 4.5)
Prothrombin time (%)	90.0 (80.2, 98.0)
Platelet count (×10^4^/mm^3^)	16.6 (11.8, 21.1)
SMI (kg/m^2^, male)	7.50 (6.97, 7.98)
SMI (kg/m^2^, female)	5.92 (5.46, 6.46)
Physical component summary score	51.1 (41.6, 54.3)
Mental component summary score	52.3 (44.2, 58.7)
AST (IU/L)	27 (21, 40)
ALT (IU/L)	24 (16, 41)
HbA1c (NGSP)	5.7 (5.4, 6.0)
eGFR (ml/min/1.73 m^2^)	82 (71, 95)
Alpha-fetoprotein (ng/mL)	3.4 (2.3, 5.8)
BTR	5.64 (4.31, 6.835)

AST, aspartate aminotransferase; ALT, alanine aminotransferase; BTR, branched-chain amino acid to tyrosine ratio; eGFR, estimated glomerular filtration rate; HBV, hepatitis B virus; HCV, hepatitis C virus; IQR, interquartile range; LC, liver cirrhosis; NBNC, non-B and non-C; NGSP, National Glycohemoglobin Standardization Program; SMI, skeletal muscle index.

**Table 2:** Univariate and multivariate analyses of factors contributing to the incidence of composite hepatic events (cutoff value for GS decrease in men, 26 kg).

Variable	Univariate	Multivariate Analysis		
	*p* Value	HR	95% CI	*p* Value
Age ≥64 years	<0.0001	1.160	0.898–1.497	0.2559
Sex	0.1137			
Body mass index ≥22.6 kg/m^2^	0.5752			
GS decrease	0.0057	1.413	1.025–1.920	0.0350
SMI decrease	0.0982			
Total bilirubin ≥0.8 mg/dL	0.0232			
Serum albumin ≥4.2 g/dL	<0.0001	0.969	0.748–1.251	0.8081
Prothrombin time ≥90.0%	<0.0001	0.740	0.564–0.970	0.0293
Platelet count ≥16.6 × 10^4^/mm^3^	<0.0001	0.994	0.760–1.299	0.9628
PCS ≥51.1	0.0318			
MCS ≥52.3	0.7995			
AST ≥27 IU/L	0.0008	1.190	0.934–1.515	0.1582
ALT ≥24 IU/L	0.0494			
Alpha fetoprotein ≥3.4 ng/mL	0.1106			
HbA1c (NGSP) ≥5.7	0.0868			
eGFR ≥82 mL/min/1.73 m^2^	0.3744			
BTR ≥5.64	<0.0001	0.988	0.770–1.271	0.9234

ALT, alanine aminotransferase; AST, aspartate aminotransferase; BTR, branched-chain amino acid to tyrosine ratio; CI, confidence interval; eGFR, estimated glomerular filtration rate; GS, grip strength; HR, hazard ratio; MCS, mental component summary score; NGSP, National Glycohemoglobin Standardization Program; PCS, physical component summary score; SMI, skeletal muscle mass index.

**Table 3:** Multivariate analyses of factors contributing to the incidence of composite hepatic events (cutoff value for GS decrease in men, 28 kg).

Variable	Multivariate Analysis		
	HR	95% CI	*p* Value
Age ≥64 years	1.165	0.902–1.504	0.2424
GS decrease	1.304	1.006–1.604	0.0460
Serum albumin ≥4.2 g/dL	0.954	0.737–1.233	0.7208
Prothrombin time ≥90.0%	0.742	0.566–0.973	0.0310
Platelet count ≥16.6 × 10^4^/mm^3^	0.999	0.766–1.305	0.9952
AST ≥27 IU/L	1.173	0.920–1.493	0.1972
BTR ≥5.64	0.967	0.754–1.244	0.7946

AST, aspartate aminotransferase; BTR, branched-chain amino acid to tyrosine ratio; CI, confidence interval; GS, grip strength; HR, hazard ratio.

## Abbreviations

ALT	alanine aminotransferase
AWGS	Asian Working Group for Sarcopenia
BIA	bioimpedance analysis
BP	bodily pain
CHEs	composite hepatic events
CI	confidence interval
CLDs	chronic liver diseases
GH	general health perception
GS	grip strength
HCC	hepatocellular carcinoma
HCV	hepatitis C virus
HE	hepatic encephalopathy
HR	hazard ratio
LC	liver cirrhosis
MELD	Model for End-Stage Liver Disease
IQR	interquartile range
MCS	mental component summary score
MH	mental health
PCS	physical component summary score
PF	physical functioning
PT	prothrombin time
QOL	quality of life
RE	role emotion
RP	role physical
SF	social functioning
SF36	36-Item Short-Form Health Survey
SMI	skeletal muscle mass index
VT	vitality
